# Attention Fusion for One-Stage Multispectral Pedestrian Detection

**DOI:** 10.3390/s21124184

**Published:** 2021-06-18

**Authors:** Zhiwei Cao, Huihua Yang, Juan Zhao, Shuhong Guo, Lingqiao Li

**Affiliations:** 1 School of Artificial Intelligence, Beijing University of Posts and Telecommunications, Beijing 100876, China; czw@bupt.edu.cn (Z.C.); gsh@bupt.edu.cn (S.G.); 2 China Mobile Research Institute, Beijing 100053, China; zhaojuan@chinamobile.com; 3 School of Computer Science and Information Security, Guilin University of Electronic Technology, Guilin 541004, China; 54pe@163.com

**Keywords:** convolution neural network, multispectral pedestrian detection, attention fusion, one-stage

## Abstract

Multispectral pedestrian detection, which consists of a color stream and thermal stream, is essential under conditions of insufficient illumination because the fusion of the two streams can provide complementary information for detecting pedestrians based on deep convolutional neural networks (CNNs). In this paper, we introduced and adapted a simple and efficient one-stage YOLOv4 to replace the current state-of-the-art two-stage fast-RCNN for multispectral pedestrian detection and to directly predict bounding boxes with confidence scores. To further improve the detection performance, we analyzed the existing multispectral fusion methods and proposed a novel multispectral channel feature fusion (MCFF) module for integrating the features from the color and thermal streams according to the illumination conditions. Moreover, several fusion architectures, such as Early Fusion, Halfway Fusion, Late Fusion, and Direct Fusion, were carefully designed based on the MCFF to transfer the feature information from the bottom to the top at different stages. Finally, the experimental results on the KAIST and Utokyo pedestrian benchmarks showed that Halfway Fusion was used to obtain the best performance of all architectures and the MCFF could adapt fused features in the two modalities. The log-average miss rate (MR) for the two modalities with reasonable settings were 4.91% and 23.14%, respectively.

## 1. Introduction

Pedestrian detection is an indispensable part of automatic driving [1]. In recent years, pedestrian detection technology, especially visible light camera-based pedestrian detection [2,3,4,5,6,7,8,9,10] using deep convolutional neural networks, has developed rapidly, and the performance of pedestrian detectors has been greatly improved. However, in the actual driving scene, the surrounding environment is complex. There are differences in the surrounding buildings, traffic signs, and background objects of different traffic. Seasonal changes will also lead to significant changes in the road scene, and the most significant change in the road scene is the lighting. In the daytime, buildings and trees may cause partial shadows. In rainy days and haze days, the brightness of the overall environment will decrease. At night, due to the lack of light, pedestrians almost blend into the background, and due to poor lighting conditions, about 70% to 75% of traffic accidents occur at night [11].

The detector based on visible-light image is usually only suitable for the road scene with sufficient illumination, and it cannot effectively detect pedestrians at night. Far infrared (FIR) camera also plays an important role in pedestrian detection, especially at night or under insufficient light conditions. The effective wavelength range of FIR camera is 6–15 m, while the wavelength of heat emitted by human body is ~9.3 m [12]. At night, pedestrians are brighter than the surrounding environment in the image. This shows that FIR image has the advantage of separating pedestrians from the surrounding environment and is suitable for pedestrian detection at night or under insufficient light, so it can be used as a supplement to visible light sensor.

Therefore, the research of pedestrian detection based on far infrared image is the key module to realize all-weather vehicle driving assistance system. At present, advanced pedestrian detection methods are based on deep neural network. Nevertheless, pedestrian detection still has great challenges, such as poor visibility, insufficient illumination conditions, and nighttime.

**Figure 1:**
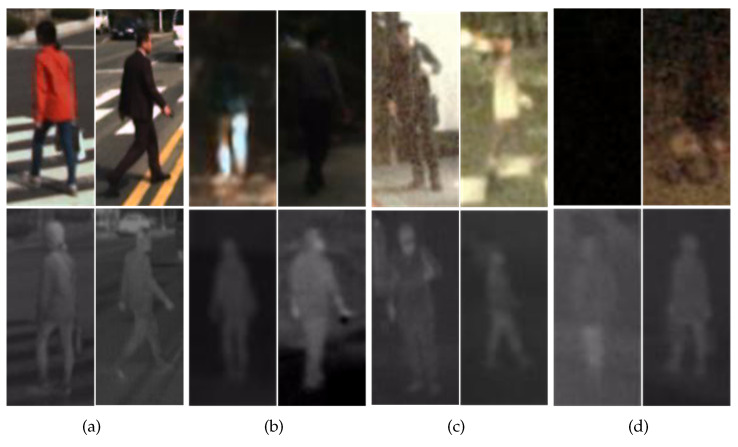
Examples of multispectral pedestrian detection images from KAIST. The first and second rows are color and thermal images, respectively. Panels (**a**,**b**) were captured in good and weak conditions in daytime traffic scenes. Panels (**c**,**d**) were captured under good and weak conditions in nighttime traffic scenes.

To overcome these problems, multispectral pedestrian detectors, which take aligned color–thermal image pairs as input, are proposed. Color images have more detailed information under good lighting conditions than thermal images, such as information on edge, texture, and color. However, when color images are captured under weak illumination conditions or during the nighttime, the feature information is lost and the detection performance is reduced; examples can be found in the first row of Figure 1. Thermal sensors are not sensitive to light and can capture pedestrians’ contours under the insufficient lighting conditions [13]; examples can be found in the second row of Figure 1. However, thermal images also have the disadvantage of lacking texture and color information. Thus, it is helpful to fuse the color and thermal images to address the challenge of insufficient illumination. Fusing color images and thermal images can be used to enrich the image information, reduce the miss rate of pedestrian detection, and improve the robustness of the model under various lighting conditions. All of these are reflected in the published KAIST [14] and Utokyo [15] multispectral pedestrian detection benchmarks and in state-of-the-art multispectral detectors [16,17,18].

Multispectral pedestrian detection methods can be divided into two-stage [16,17] and one-stage methods [18,19,20] according to the number of detection stages. In two-stage detection, the first stage is used to generate proposals, and the second stage is used to classify proposals to decide whether they are pedestrians or background. At present, the most representative multispectral pedestrian detection methods [16,17] are based on fast-RCNN [3]. They have achieved remarkable results, but the detection process is complex, and anchors must be designed with hyperparameters (anchors are manually predefined with multiple scales, ratios, and parameters), which can easily cause errors in detection. In one-stage detection, the final detection results can be obtained with only one step; One-stage detectors are more efficient owing to straightforward architectures compare with two-stage detectors. They usually adopt a straightforward fully convolutional architecture, and directly predict bounding boxes with confidence scores at each spatial position through CNNs. The bounding boxes are obtained by regression based on predefined anchors or center point of positive regions. Compared with two-stage pedestrian detection, the inference speed of one-stage is faster. The most common examples of one-stage multispectral object detectors are GFD-SSD [19], Fusion CSPNet [18], and RetinaNet [21].

Regardless of if one-stage or two-stage multispectral pedestrian detection is used, multimodal information fusion runs through the whole detection stage. Features at different modalities and different stages have different expressive abilities. The most commonly used fusion method in multispectral pedestrian detection is the network-in-network (MIN) method [22]. MIN is used to fuse features from different modalities and reduce the dimensions of multimodal features with a 1 × 1 convolution layer after concatenation. Therefore, the questions of how and where fusion takes place are two important problems in the backbone of multispectral detection. Inspired by the above two problems, we propose a novel multispectral pedestrian detection architecture with an attention fusion method to fuse color and thermal streams to detect pedestrians under weak illumination conditions. Here, we introduce a state-of-the-art one-stage object detection method, YOLOv4 [23], which can achieve real-time, high-quality, and convincing object detection results. Based on YOLOv4, we propose a novel multispectral pedestrian detection architecture for extracting features from the color and thermal streams to improve the performance. At the same time, in order to further improve the fusion of image data with different modalities, we propose a cross-modality multispectral channel attention fusion mechanism to fuse the image features between the color and thermal streams.

In this paper, the main contributions can be summarized as follows. First, we propose a one-stage multispectral pedestrian detection architecture based on YOLOv4 [23]. It merges the color and thermal streams to generate a new fusion stream to extract features and transfer the fusion information from the bottom to the top at different stages. Second, we propose a novel multispectral channel feature fusion module to integrate the features from the color and thermal streams according to the illumination conditions. Third, our method achieves state-of-the-art performance on the KAIST and Utokyo benchmarks. Our model can significantly reduce the missing of the baseline, and it provides the best performance for multispectral pedestrian detection.

The rest of this paper is organized as follows. In Section 2, related work is briefly reviewed. In Section 3, our proposed fusion architectures and fusion method, MCFF, are described in detail. In Section 4, the experimental results and an analysis are presented. Finally, conclusions and future work are summarized in Section 5.

## 2. Related Work

### 2.1. Convolution Neural Networks

Convolution Neural Network [24] is similar to multilayer perceptron, and it is a subset of deep learning and is more often utilized for classification [25] and computer vision [26] tasks. Convolutional neural network is distinguished from other neural networks by their superior performance with image, speech, or audio signal inputs. It has three main types of layers: convolutional layer, pooling layer, and fully connected (FC) layer.

The convolutional layer is the first layer of a convolutional network. While convolutional layers can be followed by additional convolutional layers or pooling layers, the fully connected layer is the final layer. With each layer, the CNNs increases in its complexity, identifying greater portions of the image. Earlier layers focus on simple features, such as colors and edges. As the image data progresses through the layers of the CNNs, it starts to recognize larger elements or shapes of the object until it finally identifies the intended object. The convolutional layer is the core building block of CNNs, and it is where the majority of computation occurs. It requires a few components, which are input data, a filter, and a feature map. Let us assume that the input will be a color image, which is made up of a matrix of pixels in 3D. This means that the input will have three dimensions—a height, width, and depth, which correspond to RGB in an image. We also have a feature detector, also known as a kernel or a filter, which will move across the receptive fields of the image, checking if the feature is present. This process is known as a convolution. After each convolution operation, a CNN applies a Rectified Linear Unit (ReLU) transformation to the feature map, introducing nonlinearity to the model.

As we mentioned earlier, another convolution layer can follow the initial convolution layer. When this happens, the structure of the CNN can become hierarchical as the later layers can see the pixels within the receptive fields of prior layers. As an example, let us assume that we are trying to determine if an image contains a bicycle. You can think of the bicycle as a sum of parts. It is comprised of a frame, handlebars, wheels, pedals, etc. Each individual part of the bicycle makes up a lower-level pattern in the neural net, and the combination of its parts represents a higher-level pattern, creating a feature hierarchy within the CNNs.

Pooling layers, also known as downsampling, conducts dimensionality reduction, reducing the number of parameters in the input. Similar to the convolutional layer, the pooling operation sweeps a filter across the entire input, but the difference is that this filter does not have any weights. Instead, the kernel applies an aggregation function to the values within the receptive field, populating the output array.

The name of the fully connected layer aptly describes itself. As mentioned earlier, the pixel values of the input image are not directly connected to the output layer in partially connected layers. However, in the fully connected layer, each node in the output layer connects directly to a node in the previous layer.

This layer performs the task of classification based on the features extracted through the previous layers and their different filters. While convolutional and pooling layers tend to use ReLu functions, FC layers usually leverage a softmax activation function to classify inputs appropriately, producing a probability from 0 to 1.

### 2.2. Multispectral Pedestrian Detection

Multispectral pedestrian detection is an important part of pedestrian detection, and many remarkable results have been achieved. According to the number of detection steps, the existing multispectral pedestrian detection methods can be divided into two-stage [14,16,17,27,28] and one-stage [18,19] methods.

Two-Stage Methods: In 2015, the KAIST multispectral pedestrian benchmark [14] was released to help detectors detect pedestrians in challenging illumination conditions, and it consisted of color–thermal image pairs. At the same time, aggregated channel features (ACFs) [2] and Adaboost were used to verify the dataset. In 2016, Wagner [29] and Liu [22] proposed a feature fusion and multispectral pedestrian detection method based on CNNs for the first time, and they used Fast-RCNN as the detection framework, which greatly improved the detection performance. In 2017, Daniel [30] presented a multispectral RPN for generating proposals and used a boosted decision tree classifier for classification. In 2018, Li [17] proposed a two-stage multispectral detection architecture based on Fast-RCNN, which combined detection and segmentation to improve detection performance. In 2019, Li [28] proposed a new fusion method to learn weights of different modalities according to the illumination conditions, and this was used to obtain detection results based on Fast-RCNN. Guan [27] also presented a multispectral pedestrian detection framework based on Fast-RCNN and joined illumination weight learning and semantic segmentation together to improve the pedestrian detection performance.

One-Stage Methods: In 2017, Hou [31] proposed a pixel-level image fusion method based on a CNN for feature fusion and used an anchor-based single-shot detector (SSD) method for pedestrian detection. In 2019, Zheng [19] used gated fusion units (GFUs) in the middle layer of an SSD for feature fusion and pedestrian detection. In 2020, Alexander [18] adopted the center and scale prediction network (CSPNet) and proposed a one-stage anchor-free multispectral fusion approach called Fusion CSPNet to fuse features from different modalities for multispectral pedestrian detection and data augmentation techniques are also used to focus on small-scale pedestrian scenes and partially occluded instances.

### 2.3. Multispectral Fusion Framework

According to the stage of fusion, CNN-based multispectral fusion frameworks can be divided into two categories: feature-level fusion [18] and decision-level fusion [28]. In the feature-level fusion, feature fusion is usually used for feature extraction. The commonly used feature fusion frameworks are Early Fusion, Half Fusion, and Late Fusion. Liu [22] implemented Early Fusion, Half Fusion, and Late Fusion based on an RCNN detection framework, and the results showed that the Half Fusion framework was obviously better than the other frameworks based on RCNNs. Li [17] and Lu [32] presented a Half Fusion framework on a Fast-RCNN for multispectral pedestrian detection to improve the detection performance. Zheng [19] proposed Stack Fusion, Gated Fusion, and Mixed Fusion based on SSD for multispectral pedestrian detection; the experimental results showed that the performance of the Gated Fusion feature fusion architecture was the best. Wolpert [18] introduced several multispectral feature fusion architectures according to the different fusion stages based on CSPNet. Finally, it was found that the Late Fusion architecture had the best detection performance.

In decision-level fusion, fusion usually appears in the final prediction stage. In general, illumination information is utilized to learn fusion weights, which are used to fuse the learning results from the color and thermal branches. Guan [27] and Li [28] proposed the fusion of the scores and bounding boxes of predictions at the decision level.

### 2.4. Feature Fusion Methods

Multispectral feature fusion is used to integrate the features from color and thermal images, and it is one of the most important ways of improving pedestrian detection performance. There are many different multispectral feature fusion methods [17,18,22,32]. In 2016, the network-in-network (MIN) method [22] was used to fuse features from different modalities and reduce feature dimensions. MIN was used to reduce the dimensions of multimodal features with a 1 × 1 convolution layer after concatenation; then, this fusion method was used in multispectral detection methods [17,18,32]. In 2018, the SUM [33] fusion method was proposed to integrate the two convolutional layers in order to detect pedestrians even in adverse illumination conditions. SUM means that the fusion features are obtained with an element-wise sum, which can be considered as the linear feature fusion with the same weight. In 2019, the illumination-aware method [27,28] was used to learn weights from illumination conditions for feature fusion. Zheng [19] proposed the GFU to learn and assign the weights of feature maps between two modalities with an SSD.

## 3. Our Approach

In this section, our proposed multispectral pedestrian detection approach is introduced, including the multispectral channel feature fusion module and multispectral fusion architecture. We first introduce YOLOv4 for multispectral pedestrian detection, then several multispectral fusion architectures for transferring the fusion information from the bottom to the top at different stages; final, the multispectral channel feature fusion method is proposed to fuse the features at different modalities.

### 3.1. YOLOv4 for Multispectral Pedestrian Detection

Based on the number of detection stages, object detection methods can be divided into one-stage and two-stage methods. One-stage detectors are more efficient because of their straightforward architectures, but two-stage detectors have advantages in accuracy. However, with the development of object detection technology, the accuracy gap between one-stage and two-stage detectors is decreasing. Here, we introduced a state-of-the-art object detection method, YOLOv4 [23], and adapted it for multispectral pedestrian detection.

**Figure 2:**
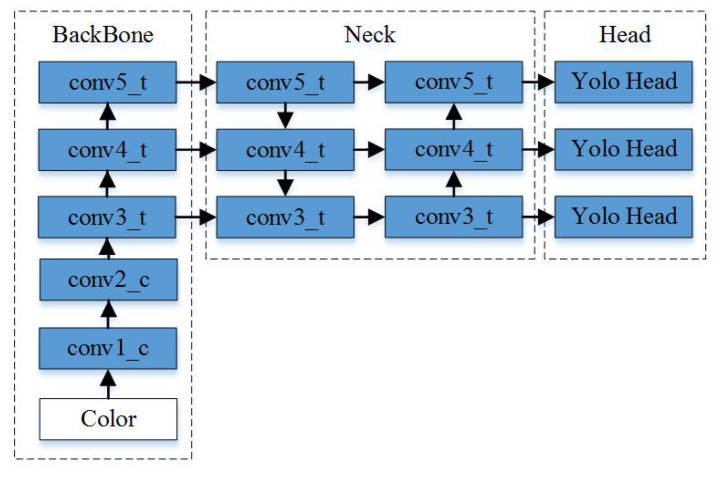
Overall object detection architecture of YOLOv4.

YOLOv4 is a one-stage anchor-based object detector that has been widely used for real-time detection. It can directly obtain detection results by predicting bounding boxes with objectness scores and classes; Figure 2 illustrates the details of the architecture. The bounding boxes are predicted by using dimension clusters as anchor boxes at three different scales. The objectness score for each bounding box is predicted by using logistic regression. Multilabel classification is used to predict the classes for each box. The architecture consists of three modules: data augmentation, feature extraction, and detection head.

The data augmentation module was used to increase the variability of the input images so that the detector had higher robustness to the images obtained from different environments. Mosaic and self-adversarial training were introduced to provide images for data augmentation.

The feature extraction module used CNNs to extract feature maps for pedestrian detection. In this detection method, CSPDarknet-53 was used as the backbone, and its convolution layers were divided into five stages according to the feature size. Given an input image of size H × W, the sizes of the three layers before the heads were $\frac{H}{32}\times \frac{W}{32}$, $\frac{H}{16}\times \frac{W}{16}$, and $\frac{H}{8}\times \frac{W}{8}$, respectively. A spatial pyramid pooling (SPP) module was added to the backbone to enhance the receptive field, and a path aggregation network (PAN) was used as the detection neck to aggregate features from different backbone levels in order to enhance the expressive abilities of the features.

The detector head modules were used to generate bounding boxes at three different scales. For each head, the bounding boxes were predicted by using dimension clusters as anchor boxes, and complete intersection over union (CIoU) [34] was used as the loss function for regression. The objectness score for each bounding box was predicted through logistic regression. Multilabel classification was used to predict the classes for each box.

### 3.2. Fusion Architectures

**Figure 3:**
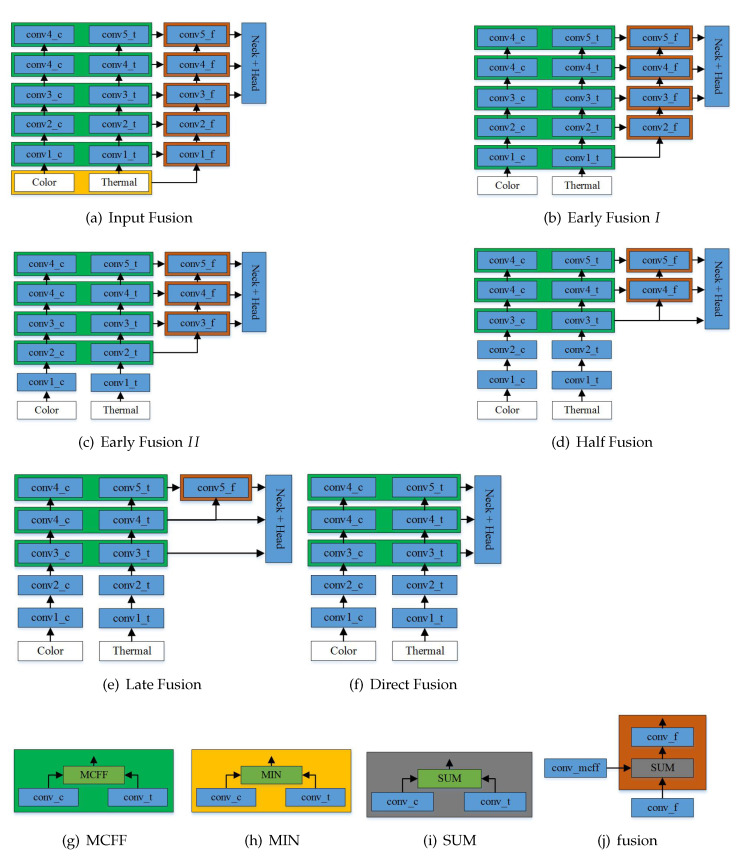
An overview of the fusion architectures. (**a**–**f**) Six fusion architectures that we proposed. (**g**–**i**) Fusion methods used in panels (**a**–**f**). The green, yellow, and black fusion blocks are the MCFF, MIN, and SUM, respectively. (**j**) was used to fuse features from the MCFF and $conv_{-}f$.

The current multispectral fusion architectures [16,18,28,35] fuse different branches into one and lack the information transmission from the bottom to top between each mode. We designed a novel feature fusion architecture based on color and thermal streams to extract features. It fuses the color and thermal streams to generate a new fusion stream to extract features and transfer the fusion information from the bottom to the top at different stages. For the convenience of illustration, the backbone can be divided into five blocks, which were named $conv1_{-}x$, $conv2_{-}x$, $conv3_{-}x$, $conv4_{-}x$, and $conv5_{-}x$ according to the feature map size. The MCFF was used to fuse the features $conv_{-}c$ in the color stream and $conv_{-}t$ in the thermal stream in order to obtain the new branch $conv_{-}f$ for pedestrian detection. At the beginning of the multispectral feature fusion stage, the fusion approach can be divided into Input Fusion, Early Fusion *I*, Early Fusion II, Halfway Fusion, Later Fusion, and Direct Fusion. An overview of the architectures is shown in Figure 3.

Input Fusion is data fusion before feature extraction. MIN is used to stack the color and thermal images in the channel dimension and then reduce the channel dimensions of the multi-modality color–thermal images to those of the single-color-modality images for the input, as shown in Figure 3a. The MCFF is used to fuse the features at the stage behind the input layer.

Early Fusion *I* fuses the features after the first convolutional block conv1; the details can be found in Figure 3b. The MCFF is used to fuse the features from $conv1_{-}c$ in the color stream and $conv1_{-}t$ in the thermal stream, and this reduces the dimensions of the fusion layer to 256, similarly to the input of the new fusion branch $conv_{-}f$. The Early Fusion model fuses features at a low level.

Early Fusion II fuses the features after the second convolutional block conv2 for the new branch. The MCFF is also used to fuse the features from $conv2_{-}c$ in the color image branch and $conv2_{-}t$ in the thermal image branch, as shown in Figure 3c.

Halfway Fusion starts fusion in the middle stage of feature extraction, as shown in Figure 3d. Differently from Early Fusion, it starts to fuse both streams after the third convolutional block conv3. The MCFF is also used after the concatenation layer for the same reasons as those discussed before. The features from the conv4 layers contain more semantic meanings than the conv1 features do, though they retain some fine visual details. This fusion architecture was selected as the baseline because of its excellent performance in the experiments.

Late Fusion performs feature fusion at the end stage, as shown in Figure 3e. It is a kind of high-level fusion; only block $conv5_{-}f$ is used to transfer information. Fused features from conv3, conv4, and $conv5_{-}f$ are used to detect pedestrians.

Direct Fusion has the same fused architecture as that of Fusion CSPNet [18] but has a different fusion method. The MCFF is introduced instead of MIN to balance the features from both streams. There are no new streams for fusing the color and thermal streams at different stages. The features at the different modalities are directly fused from stages 3, 4, and 5. This can be seen in Figure 3f.

### 3.3. Multispectral Channel Feature Fusion

In multispectral pedestrian detection, the fusion of data with different modalities is a very important step. An efficient fusion method should be able to supervise the information fusion of different modalities and improve the performance of the detector. The existing multispectral feature fusion methods are mainly the SUM and MIN methods [30]. The SUM operator denotes an element-wise sum of features, and it can be considered as linear feature fusion with the same weight. The MIN method is used to reduce the dimensions of multi-modal features with a 1 × 1 convolution kernel after concatenation; this is unsupervised nonlinear feature fusion. Therefore, we have a reason to design a feature fusion method that uses the care condition as the supervision condition.

**Figure 4:**
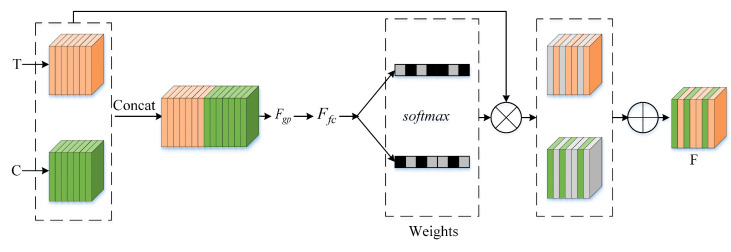
Illustration of multispectral channel feature fusion. The first stage of the MCFF concatenates features from the color and thermal modalities in the channel dimension. Then, the second stage uses the learning weight to aggregate features in an adaptive way.

Color and thermal images play different roles under different illumination conditions. Most pedestrians are in good lighting conditions during the daytime, except in some cases (standing in shadows); however, thermal images are not sensitive to illumination. In contrast, thermal images can capture better visual features of pedestrians at night. Therefore, it is reasonable to design a supervised feature fusion method that uses illumination; here, we introduce the MCFF, which can adaptively adjust channel features between the color and thermal modalities for fusion under different illumination conditions. The details of the MCFF can be found in Figure 4. Three steps are needed to implement the MCFF.

In the first step, the features from the color (C) and thermal (T) branches are concatenated in the channel dimension. The concatenated feature maps can be presented as

(1) $$F_{i}=\mathrm{Concat}\left(C_{i},T_{i}\right)$$

where $C_{i}$ and $T_{i}$ are defined as the color and thermal features at the *i*-th level. $C_{i},T_{i}\in R^{\frac{H}{r}\times \frac{W}{r}\times C}$. *r* is the stride, and $r=2^{i}$.

In the second step, global average pooling (GAP) and global max pooling (GMP) are used to generate the channel feature vectors $F^{1}$. The *c*-th channel element from GAP and GMP is calculated with the following formula:

(2) $$F_{c}^{1}=GAP\left(X_{c}\right)+GMP\left(X_{c}\right)$$

Then, a new compact feature $F^{2}$ is created to adaptively learn the fusion weights of the color and thermal features. This is achieved with a fully connected (FC) layer with a lower dimension of

(3) $$z=F^{2}=FC\left(F^{1}\right)$$

where $F^{1}\in R^{C}$, $F^{2}\in R^{C^{\prime }}$, $C^{\prime }=max\left(C/r,L\right)$ is the typical setting in our experiment.

Furthermore, softmax is used for normalization, and the learned weights $\alpha_{c}$ and $\beta_{c}$ are used to select the corresponding level features for the final fusion $F_{c}$. Note that $\alpha_{c}$ and $\beta_{c}$ are simply a scale value at channel *c* and $\alpha_{c}$,$\beta_{c}\in$ [0, 1].

(4) $$\alpha_{c}=\frac{e^{A_{c}z}}{e^{A_{c}z}+e^{B_{c}z}}$$

(5) $$F_{c}=\alpha_{c}\cdot C_{c}^{i}+\beta_{c}\cdot T_{c}^{i}$$

where $F_{c}\in R^{\frac{H}{r}\times \frac{W}{r}}$, $\alpha_{c}+\beta_{c}=1$, and $A,B\in R^{C\times C^{\prime }}$. With this method, the features at all of the levels are adaptively aggregated at each scale. The output of the MCFF can be used as the input of a new fusion branch for feature extraction.

For the convenience of description, we assume that multimodal feature fusion is performed in stage 3 of the backbone, and the input image size is 1×640×640×3. The feature of thermal image in stage 3 is $conv3_{-}f$ and the feature of color image in stage 3 is $conv3_{-}c$, the feature size of the two modes is 1×80×80×256. First, $conv3_{-}c$ and $conv3_{-}f$ are concatenated in channel dimension. Then, the global information of the features are obtained by GAP and GMP, FC layers are used to learn weights and extract illumination scale of each channel, the scale size is 1×1×256×2. Finally, the multimodal features are fused according to the learned scale of each channel reference to formula (5). Under sufficient illumination, the weight of color features should be relatively bigger; otherwise, under the condition of night or insufficient light, the weight of thermal features should be relatively large. According to feature fusion method in stage 3, the fusion features of different modes in stage 4 and stage 5 can be obtained. The fused feature branch also uses the same convolution module as the backbone to extract features and learn the model.

## 4. Experiments

In this section, the multispectral pedestrian dataset, evaluation metrics, and implementation details are first introduced. Then, ablation studies of the MCFF and fusion architecture are reported. Finally, we also give a detailed description of the benchmark comparison experiments.

### 4.1. Dataset and Metrics

The KAIST Multispectral Pedestrian Dataset [14] is an ego-centric moving-view traffic scene image dataset that includes different lighting conditions from day to night. Each pair of images was captured with a visible sensor (color image) and a thermal sensor (thermal image), and then aligned with a beam splitter at the pixel level. The resolution of the images is 640 × 480 px. In the training stage, a new training subset containing 25,086 images was sampled from the training videos with two-frame skips. The test set of KAIST contained 2252 images sampled from the test videos with 30-frame skips, among which 1455 images were captured during the daytime, and 797 others were captured during the nighttime. The dataset had two different annotations: original annotations [14] and improved annotations [17]. For a thorough comparison of the methods, we chose the improved annotations (${\mathrm{MR}}_{-2}^{I}$) for the evaluation.

The Utokyo dataset [15] is a multispectral dataset that was taken during both daytime and nighttime with four different cameras (RGB, FIR, MIR, and NIR) on autonomous vehicles. A total of 7512 group images were captured—3740 during the day and 3722 at night. Five common objects (bikes, cars, carstops, colorcones, people) were labeled in this dataset. It used 1466 groups of correctly aligned images with size of 320 × 256 px as a test set.

To evaluate our method, the log-average MR over the false positive per image (FPPI) in a range of [$10^{-2}$, $10^{0}$] (denoted as ${\mathrm{MR}}_{-2}$) [36] was used to measure the pedestrian detection performance. We implemented the experiments under reasonable and all setting [14]. Generally speaking, the height in the reasonable setting was greater than 55 pixels, and in the all settings, it was greater than 20 pixels.

### 4.2. Implementation Details

Our method was implemented in the same configuration as that of YOLOv4 [23]. To train with KAIST and Utokyo, the input image size was set to 640 × 640 px. If the label in the ground truth is “person” and the height was greater than 50 px, it was used in the training set. Otherwise, it was marked to be ignored. In the experiment with Utokyo, only the RGB and FIR images were used as input images for the comparison. Before training, the k-means cluster method was used to get anchors. In the experiment with KAIST, the anchors were (16,38), (22,53), (31,74), (43,102), (59,141), (82,196), (113,271), (156,375), and (216,520). In the experiment with Utokyo, the anchors were (13,24), (18,33), (24,45), (32,76), (44,106), (82,196) and (154,206), (206,324), (293,478).

At the stage of testing, the original size was used as the input to predict predict the height, offset, and location. We first selected bounding boxes with scores above 0.001, and then used non-maximum suppression (NMS) with an overlap threshold of 0.65 for the final processing.

Specifically, our multispectral pedestrian detection method was trained using the stochastic gradient descent (SGD) optimizer with an initial learning rate of 0.0001 and a learning policy for the steps. The number of training epochs was set to 100, and each mini-batch was constructed from 80 images. To carry out the experiments, an Intel Xeon E5-2620 at 2.1 GHz CPU server with 90 GB of memory and four Tesla P40 (24 GB) GPUs was used.

### 4.3. Ablation Studies

#### 4.3.1. Impacts of Fusion Architectures

To evaluate our fusion architectures, color-only and thermal-only were also implemented for a comparison. In Table 1, it can be seen that the MR values of the color-only and thermal-only detection for KAIST in the reasonable setting were 20.50% and 16.64%, and the MR values for Utokyo were 33.55% and 32.18%. It is clear that thermal-only detection was several points better than color-only detection for KAIST. This demonstrated that thermal sensors are more useful in this multispectral pedestrian dataset. Among these multimodality feature fusion architectures, Input Fusion performed worse in the reasonable setting and in all settings. The gap between single modality and multimodality was large. For KAIST, the MR with Input Fusion was 6.06%. For Utokyo, the MR of Input Fusion was 28.80%. This shows that multimodality pedestrian detection can significantly improve the detection performance.

Among these multimodality feature fusion architectures, Halfway Fusion performed better than the other fusion architectures in the reasonable setting and in all settings. For KAIST and Utokyo, the MR values of Halfway Fusion were 4.91% and 23.14%, respectively, in the reasonable setting. The gaps between Input Fusion and Halfway Fusion were about 1.15% and 5.66% for KAIST and Utokyo. This shows that low-level features may reduce the detection performance. Observing Table 1, it is clear that the performance of Direct Fusion was the second best. As displayed in Figure 3f, Direct Fusion directly extracted features at different modalities in stages 3, 4, and 5. By comparing Halfway Fusion and Direct Fusion, we can reach the conclusion that one more new fusion stream can effectively transfer information from the bottom to the top and extract features from different stages.

#### 4.3.2. Comparison of the Three Fusion Methods

In this paper, the MCFF adaptively selects the features from the color stream and thermal stream depending on the illumination conditions to detect pedestrians. In order to evaluate the effectiveness of the MCFF, we compare it with two other fusion methods—SUM and MIN—based on our proposed half-fusion architecture. KAIST and Utokyo were used to evaluate the fusion methods, which were evaluated not only in the reasonable setting, but also in all settings. The comparative miss rate results are illustrated in Table 2.

**Figure 5:**
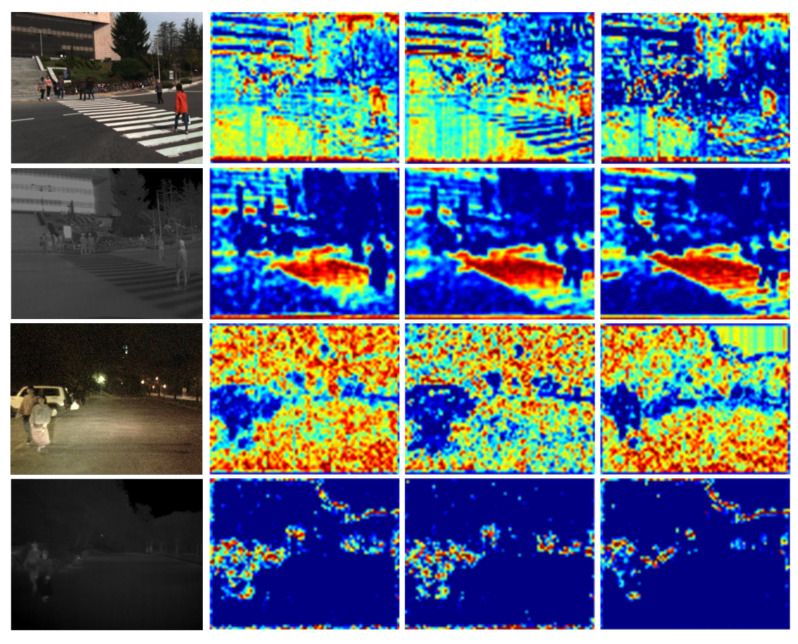
Feature map visualization in stage3 on KAIST. The first column shows the color and thermal image pairs, the second column shows feature maps after MCFF, the third column shows feature maps from color image stream, and the fourth column shows feature mapsfrom thermal image stream.

**Figure 6:**
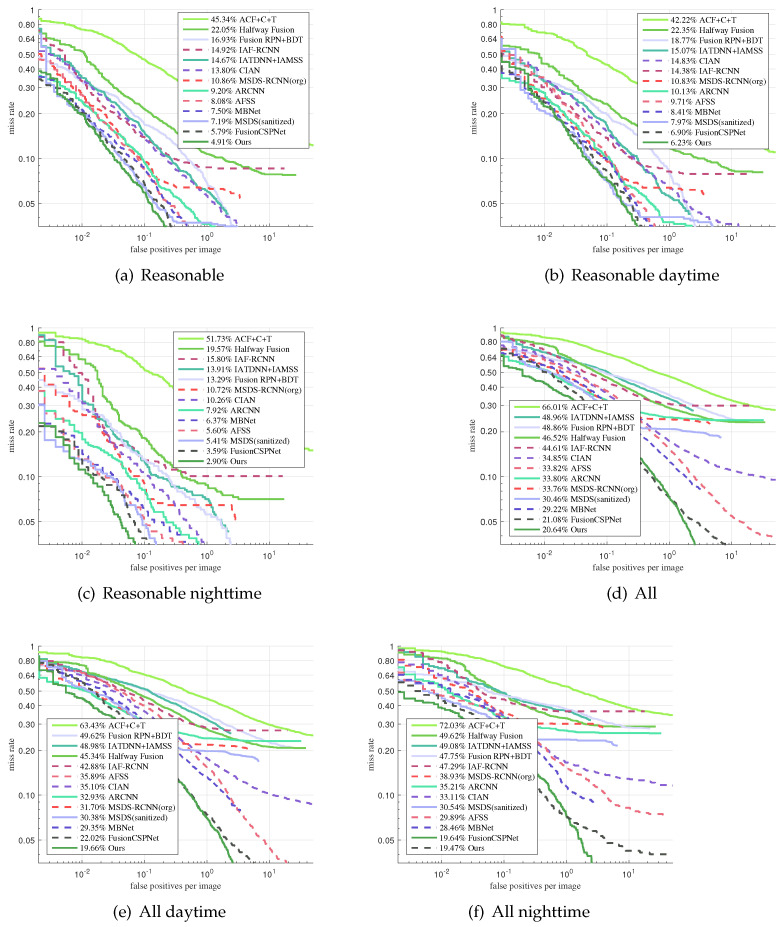
Comparison of the detection results on the KAIST dataset in reasonable and all settings: all day, daytime, or nighttime. (**a**) Reasonable all-day, (**b**) reasonable daytime, (**c**) reasonable nighttime, (**d**) all all-day, (**e**) all daytime, and (**f**) all nighttime.

From the results in Table 2, it can be seen that the MR of the MCFF on KAIST and Utokyo was the lowest among the three fusion methods in the reasonable setting. On KAIST, the performance had relative improvement rates of 14.6% and 6% compared to the other multispectral pedestrian fusion methods. The feature map in stage 3 can be found in Figure 5. We can get the information that the fused features have stronger semantic expression ability than single modal features. On Utokyo, the performance was also improved by 3% and 11% based on the half-fusion architecture. It was also noticed that the performance of the MCFF in all settings was excellent. On KAIST and Utokyo, the MCFF had the best performance compared to the other multispectral pedestrian fusion methods (SUM and MIN). Overall, our proposed MCFF had excellent feature fusion performance after the color stream and thermal stream were fully integrated. As we can see from Figure 6, the two modality feature maps are remedied with the differential information from each other.

### 4.4. Comparison with the State-Of-The-Art

**Figure 7:**
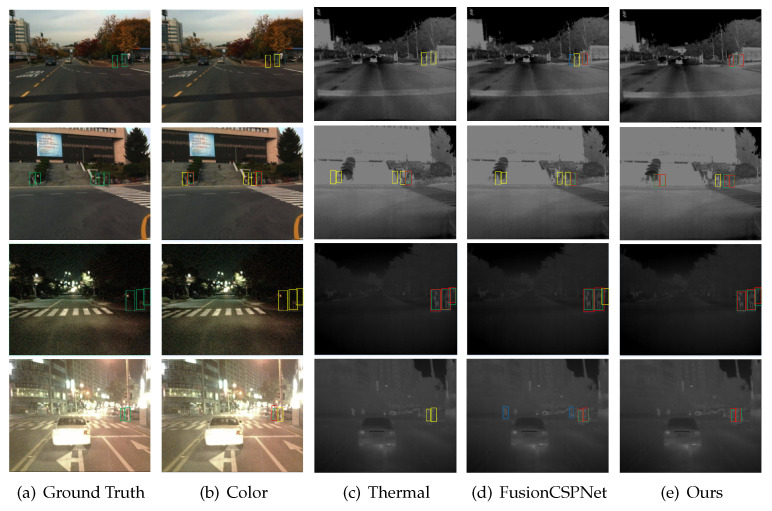
Qualitative comparison of multispectral pedestrian detection results in the KAIST reasonable test subset with other state-of-the-art approaches. (**a**) ground truth, (**b**) detection results of YOLOv4 trained by color dataset only, (**c**) detection results of YOLOv4 trained by thermal dataset only, (**d**) detection results of FusionCSPNet trained by multispectral dataset, (**e**) detection results of ours method trained by multispectral dataset. Note that green bounding boxes (BBs) show GT labels, red and blue BBs are detection result, red BBs show TP, blue BBs show FP, and yellow shows FN. Best viewed in color.

We evaluated the proposed fusion method on the KAIST testing dataset in the reasonable setting and in all settings in comparison with ACF+T+THOG [14], Halfway Fusion [22], Fusion RPN+BDT [30], IAF R-CNN [28], IATDNN+IASS [27], CIAN [37], MSDS-RCNN [17], ARCNN [38], MBNet [39], and FusionCSPNet [18]. Among these detection methods, FusionCSPNet and our method were one-stage methods, and the rest were two-stage methods. The experimental results in Figure 6a show that our detection method outperformed all of these methods and achieved the lowest MR of 4.91% under the reasonable setting. At the same time, in order to more intuitively compare the detection results of these detectors, we qualitatively evaluated the four multi-spectral pedestrian detectors under the Reasonable test subset, as shown in Figure 7. A similar trend was observed for the other reasonable day and night subsets. In Figure 6b,c, we can observe that our approach also obtained excellent performance in the reasonable setting during the daytime and nighttime. However, the performance of our approach in the nighttime was better than in the daytime. This demonstrates that our proposed detection method is more useful for pedestrian detection under dark illumination conditions.

We also found that our approach obtained the best accuracy on KAIST in all the settings, as shown in Figure 6d–f, compared with the state-of-the-art methods. This demonstrates that our method has the ability to distinguish people at all scales.

**Figure 8:**
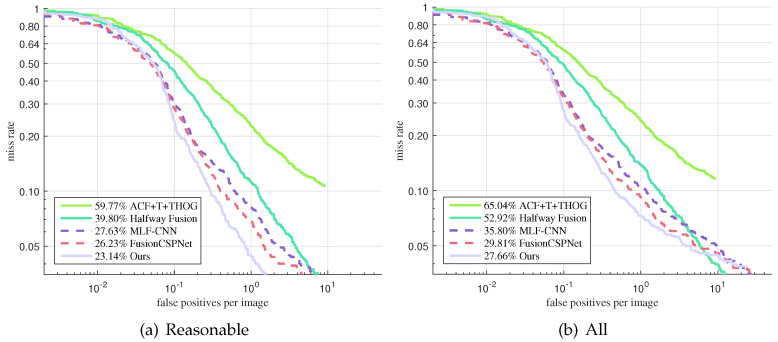
Comparison of the detection results reported on the test set of Utokyo. (**a**) detection results under reasonable setting, (**b**) detection results under all setting.

**Figure 9:**
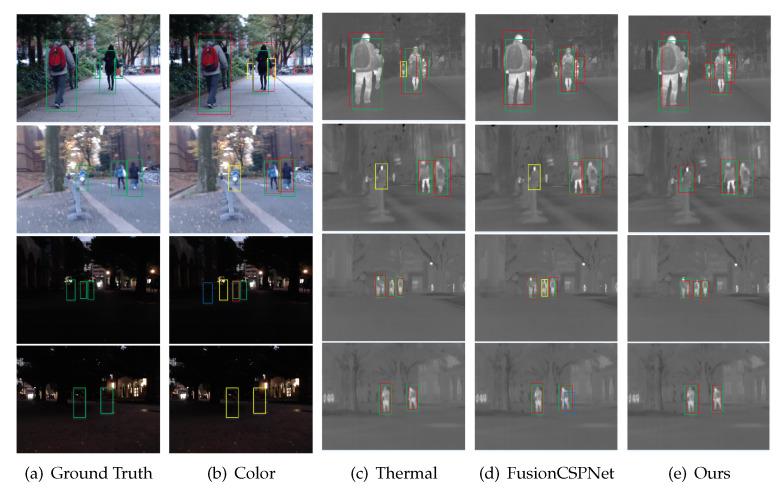
Qualitative comparison of multispectral pedestrian detection results in the Utokyo reasonable test subset with other state-of-the-art approaches. (**a**) ground truth, (**b**) detection results of YOLOv4 trained by color dataset only, (**c**) detection results of YOLOv4 trained by thermal dataset only, (**d**) detection results of FusionCSPNet trained by multispectral dataset, (**e**) detection results of ours method trained by multispectral dataset. Note that green bounding boxes (BBs) show GT labels, red and blue BBs are detection result, red BBs show TP, blue BBs show FP, and yellow shows FN. Best viewed in color.

As Figure 8 shows, we also evaluated the proposed approach on the Utokyo testing dataset in the reasonable setting and in all settings in comparison with ACF+T+THOG [14], Halfway Fusion [22], MLF-CNN [22], and FusionCSPNet [18]. ACF+T+THOG, Halfway Fusion, and MLF-CNN are two-stage detection methods; FusionCSPNet and our method are one-stage methods. FusionCSPNet is the best among the existing detectors, with a 20.61% MR under the reasonable setting and a 49.52% MR under all settings. With the proposed method, we obtained MR values of 16.93% and 44.84%, respectively, thus improving upon the current state-of-the-art by 20% and 9%. It can be observed that our approach obtained the best performance. Furthermore, we qualitatively show some sample detection results in Figure 9.

As shown in Figure 6 and Figure 8, our approach achieved remarkable performance compared with the other methods, especially the two-stage methods. These prove that our one-stage detection method is more suitable for multispectral pedestrian detection.

Table 3 illustrates the computational cost of our method compared to the state-of-the-art methods. It can be observed that the proposed multispectral detection method is also time efficient during inference stage, with only 31 ms/image on a single NVIDIA Tesla P40 GPU card. This result also shows that the runtime of one-stage multispectral detection methods is more efficient than two-stage multispectral detection methods.

## 5. Conclusions

In this paper, we focused on the task of multispectral pedestrian detection by using color and thermal image pairs. We first proposed a multispectral fusion architecture based on the one-stage object detection method YOLOv4. The proposed architecture transferred information on the fusion stream from the bottom to the top and fused the features at the different stages to improve the performance. Then, a novel multispectral attention fusion method, MCFF, was introduced to fuse the color and thermal streams according to the illumination conditions. Through experimental comparisons, we observed that the one-stage Halfway Fusion architecture had the best performance among the proposed fusion architectures. The experimental results demonstrated that the proposed fusion method with MCFF had excellent performance compared with the MIN and SUM methods. Overall, our detection method achieved state-of-the-art performance on KAIST with new annotations and on the Utokyo multispectral pedestrian dataset.

## Figures and Tables

**Table 1:** Comparison of different fusion architectures. Performance are evaluated in terms of log-average miss rate.

Architecture	KAIST		Utokyo	
	Reasonable (%)	All (%)	Reasonable (%)	All (%)
Color Only	20.50	43.06	33.55	36.74
Thermal Only	16.64	35.32	32.18	35.32
Input Fusion	6.06	23.15	28.80	32.62
Early Fusion *I*	5.99	22.50	24.21	27.51
Early Fusion II	5.65	22.25	24.91	28.82
Halfway Fusion	4.91	20.64	23.14	26.21
Late Fusion	5.35	22.07	24.86	27.93
Direct Fusion	5.21	21.25	23.49	26.78

**Table 2:** Comparison of the different fusion methods on KAIST and Utokyo.

Method	KAIST		Utokyo	
	Reasonable (%)	All (%)	Reasonable (%)	All (%)
SUM	5.75	21.50	23.69	27.05
MIN	5.22	20.94	23.20	27.26
MCFF	4.91	20.64	23.14	26.21

**Table 3:** Comparison of runtime with state-of-the-art methods.

Method	KAIST	Utokyo	Plateform
ACF [14]	2.73	-	MATLAB
Halfway Fusion [22]	0.43	-	TITAN X
Fusion RPN + BF [30]	0.8	-	MATLAB
IAF R-CNN [28]	0.21	-	TITAN X
IATDNN + IASS [27]	0.25	-	TITAN X
CIAN [37]	0.07	-	1080 Ti
MSDS-RCNN [17]	0.22	-	TITAN X
ARCNN [38]	0.12	-	1080 Ti
MBNet [39]	0.07	-	1080 Ti
FusionCSPNet [18]	0.08	0.06	P40
Ours	0.03	0.03	P40

## Data Availability

Not applicable.
